# State of the Field: Cytotoxic Immune Cell Responses in *C. neoformans* and *C. deneoformans* Infection

**DOI:** 10.3390/jof10100712

**Published:** 2024-10-12

**Authors:** Elizabeth C. Okafor, Kirsten Nielsen

**Affiliations:** 1 Department of Medicine, University of Minnesota, Minneapolis, MN 55455, USA; okafo039@umn.edu; 2 Department of Microbiology and Immunology, University of Minnesota, Minneapolis, MN 55455, USA; 3 Department of Biomedical Sciences and Pathobiology, Virginia Tech University, Blacksburg, VA 24060, USA

**Keywords:** human immunodeficiency virus (HIV), advanced immunodeficiency virus (AIDS), fungal infection, *Cryptococcus neoformans*, cryptococcal meningitis, cytotoxic cells, natural killer cells, CD8^+^ T-cells, gamma/delta T-cells, CD4^+^ T-cells

## Abstract

*Cryptococcus neoformans* is an environmental pathogen that causes life-threatening disease in immunocompromised persons. The majority of immunological studies have centered on CD4^+^ T-cell dysfunction and associated cytokine signaling pathways, optimization of phagocytic cell function against fungal cells, and identification of robust antigens for vaccine development. However, a growing body of literature exists regarding cytotoxic cells, specifically CD8^+^ T-cells, Natural Killer cells, gamma/delta T-cells, NK T-cells, and Cytotoxic CD4^+^ T-cells, and their role in the innate and adaptive immune response during *C. neoformans* and *C. deneoformans* infection. In this review, we (1) provide a comprehensive report of data gathered from mouse and human studies on cytotoxic cell function and phenotype, (2) discuss harmonious and conflicting results on cellular responses in mice models and human infection, (3) identify gaps of knowledge in the field ripe for exploration, and (4) highlight how innovative immunological tools could enhance the study of cytotoxic cells and their potential immunomodulation during cryptococcosis.

## 1. Introduction

The fungal pathogen *Cryptococcus neoformans* is ubiquitously found in the environment and pulmonary asymptomatic infection is commonly acquired during childhood [1,2,3]. Chronic immunodeficiency, such as in the case of organ transplant or human immunodeficiency virus (HIV) infection, can lead to reactivation of asymptomatic pulmonary infection and can result in dissemination into the vasculature and central nervous system (CNS) [4,5,6,7,8,9]. Persons with advanced HIV can develop a life-threatening CNS Cryptococcus infection called cryptococcal meningitis (CM), which is the most common cause of meningitis in sub-Saharan Africa [10,11,12]. The annual incidence of CM is estimated to be 152,000 cases globally; however, despite antiretroviral (ART) and antifungal therapies, the mortality rate remains around 25% with optimal care and often over 40% with routine care in low- and middle-income countries [13,14,15].

Evaluating *C. neoformans* cellular morphology through several microscopy methodologies has revealed the unique architectural structure of the dense polysaccharide capsule composed of glucuronoxylomannan (GXM), galactoxylomannan (GalXM), and mannoproteins; by contrast, the cell wall is composed mainly of α-glucan, β-glucan, mannoproteins, chitosan, and melanin [16,17,18,19,20,21]. Both the polysaccharide capsule and fungal cell wall are highly immunogenic, primarily to antigen presenting cells such as dendritic cells, macrophages, and monocytes [22,23,24]. Moreover, shedded capsule and cell wall components in the cell culture filtrate can activate effector functions of CD4^+^ T-cells and CD8^+^ T-cells [24,25].

In advanced HIV, CD4^+^ T-cells are transformed into viral replication factories and are destroyed [26,27,28]. CD4^+^ T-cells are important players in orchestrating the innate and adaptive immune responses to *C. neoformans* through co-stimulation of antigen presenting cells, cytokine and chemokine secretion, and formation of antigen specific memory cells [29,30,31,32]. The absence of functional CD4^+^ T-cells affects several types of peripheral blood mononuclear cells (PBMCs) and can impair the formation of inflammatory macrophage populations, influence neutrophil recruitment to infected tissues, and alter cytotoxic cell activation [33,34,35]. Collectively, this weakens the body’s ability to eliminate *C. neoformans* cells ultimately increasing morbidity and mortality. There are several outstanding reviews discussing CD4^+^ T-cells within the broader immune response to *C. neoformans* infection [16,30,36,37,38,39]. However, there are no detailed reviews examining in detail cytotoxic cell populations during *C. neoformans* infection and their importance in fungal clearance.

Cytotoxic cells, including CD8^+^ T-cells, Natural Killer (NK) cells, gamma/delta (γδ)T-cells, NK T-cells, and cytotoxic CD4^+^ T-cells, have the unique ability to target pathogens through antigen-dependent and/or antigen-independent processes. Cytotoxic cells express a repertoire of activating and inhibitory receptors which facilitate or inhibit cytotoxic function, respectively. CD8^+^ T-cells, NK cells, γδ T-cells, NK T-cells, and cytotoxic CD4^+^ T-cells differ based on receptor expression and utilize the T-cell receptor, natural cytotoxicity receptors (NCRs), C-type lectin-like receptor family (NKG2 family), and killer-like (KIRs) receptors to recognize host cell damage- and pathogen-associated molecular patterns [40,41,42,43,44]. Additionally, NK cells can recognize antibody-opsonized pathogens or infected host cells through engagement of FcγRIIIa (CD16A) on the cell surface and the Fc region of antibodies, a mechanism called antibody mediated cellular cytotoxicity (ADCC) [45]. Cumulative engagement of these receptors triggers intracellular signaling cascades and the fusion of cytolytic granules to the cell membrane. The release of pro-apoptotic molecules, such as granzymes, granulysin, FasL, and perforin, into the tissue environment induces apoptosis of target cells [46,47].

In this review, we summarize the current body of literature regarding cytotoxic cells during *C. neoformans* and *C. deneoformans* pulmonary and CNS disease, noting both fungal strain and clinical isolate used in the various studies. We provide a comparison between mouse and human studies, highlighting current gaps in knowledge within the field that are optimal for future pursuit. We discuss cytotoxicity mechanisms of CD8^+^ T-cells and NK cells against *C. neoformans* and *C. deneoformans*. Lastly, we report on innovative immunological tools being introduced into the Cryptococcus field.

## 2. CD8^+^ T-Cells in the Adaptive Immune Response

### 2.1. Murine Localized Pulmonary Infection

**Figure 1:**
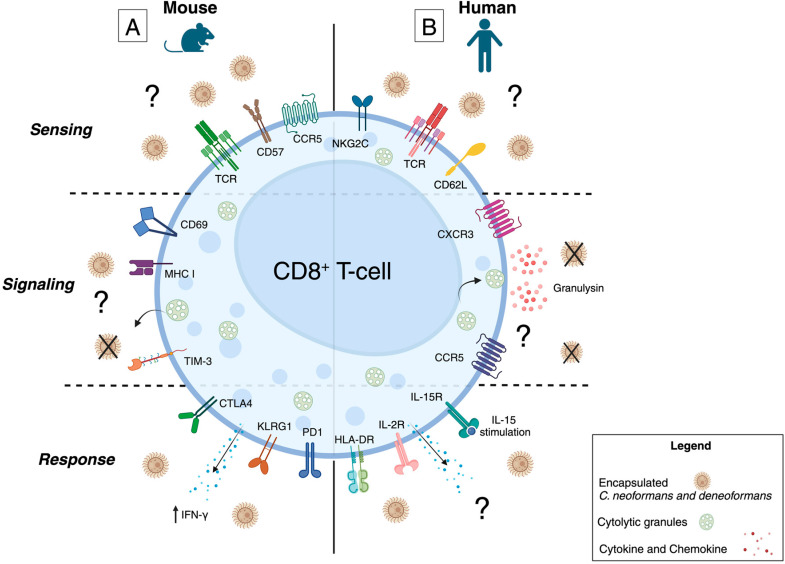
Murine and Human CD8^+^ T-cell Sensing, Signaling, and Response to *C. neoformans* and *C. deneoformans.* Summary of the current body of literature regarding the mechanism of (**A**) murine and (**B**) human CD8^+^ T-cell targeting, degranulation, and cytokine secretion after exposure to *C*. *neoformans* or *deneoformans.* (**A**) Despite depletion of murine CD8^+^ T-cells in the lung and systemic circulation leading to increased CFUs, the exact receptor(s) and fungal ligand(s) which trigger cytotoxicity and the cytolytic proteins secreted are unknown. During infection, infiltrating pulmonary and systemic CD8^+^ T-cells are robust producers of IFN-γ. (**B**) Similar to murine CD8^+^ T-cells, the exact receptor(s) and fungal ligand(s) which facilitate cytotoxicity of human CD8^+^ T-cells are unknown. Upon exposure to fungal cells, human CD8^+^ T-cells increase transcription of the cytolytic protein granulysin and depletion of granulysin abrogates CD8^+^ T-cell anti-cryptococcal effects. The mechanism by which granules containing granulysin are selectively secreted, over those containing perforin or other cytolytic molecules, remains to be determined. IL-15 stimulation enhances human CD8^+^ T-cell anti-cryptococcal effects while IL-2R expression increases upon coculture with fungal cells. However, the cytokines released by activated CD8^+^ T-cells during human cryptococcal meningitis have not been identified.

During murine pulmonary *C. deneoformans* (52D) infection, CD8^+^ T-cells are recruited to the lung and lung-associated lymph nodes (LALN) [31,48,49]. By week two of infection, the infiltrating pulmonary CD8^+^ T-cell expression profile consists of CD69^+^ IL-2R/CD25^+^ CD62L^low^ CD44^low^, denoting active cells with residency and proliferation potential [31,48]. Upon ex vivo stimulation with anti-CD3 and anti-CD28 or with *C. deneoformans* (52D) lysate, infiltrating pulmonary CD8^+^ T-cells produce more interferon (IFN)-γ when compared to CD8^+^ T-cells isolated from the LALN. This result indicates that pulmonary CD8^+^ T-cells sense and recognize fungal antigens and retain their key ability to produce the inflammatory cytokine IFN-γ [31] (Figure 1A).

To further investigate the relationship between CD4^+^ and CD8^+^ T-cells, depletion experiments were performed in mice. Recruitment of CD8^+^ T-cells to the lung is maintained in the context of CD4^+^ T-cell depletion, suggesting that the presence of CD4^+^ T-cells is not required for trafficking of CD8^+^ T-cells to the infected lung [48]. Consistent with previous results, the infiltrating pulmonary CD8^+^ T-cells, in CD4^+^ T-cell-depleted mice, express IFN-γ upon ex vivo stimulation but not tumor necrosis factor (TNF)-α, another important inflammatory cytokine [48,49]. Blocking IFN-γ by neutralizing antibody led to a significant increase in pulmonary colony forming units (CFUs) and the number of macrophages with intracellular fungal cells in the CD4+ T-cell-depleted mice [48]. This outcome implies that pulmonary macrophages are unable to destroy *C. deneoformans* (52D) in the phagosome without sufficient IFN-γ in the tissue environment. Thus, CD8^+^ T-cell IFN-γ expression is crucial for controlling pulmonary fungal dissemination during both wildtype and CD4^+^ T-cell depletion models of pulmonary infection.

Alongside CD4^+^ T-cell depletion models, IFN-γ production has been studied in two additional mouse models. In the context of IL-17^−/−^ pulmonary *C. deneoformans* (52D) infection, CD8^+^ T-cells produced less IFN-γ compared to wildtype mice at one- and two-weeks post infection; however, IFN-γ expression was comparable by four weeks post infection [50]. In mice with impaired Notch signaling, there was no difference in the number of CD8^+^ T-cells recruited to the lung four weeks post pulmonary *C. deneoformans* (52D) infection, and the activation and memory status, per CD44 and CD62L expression, respectively, was similar to wildtype infected mice [51]. However, upon ex vivo stimulation with anti-CD3 and anti-CD28, there were significantly fewer infiltrating pulmonary CD8^+^ T-cells producing IFN-γ compared to wildtype [51]. These data suggest that potentially several pathways, including IL-17 and Notch signaling, are involved in IFN-γ production by infiltrating pulmonary CD8^+^ T-cells at various timepoints post-infection.

Recent studies of latent infections in immunocompetent mice also show a potential role for pulmonary infiltrating CD8^+^ T-cells. In a latent infection with a Uganda clinical isolate (UgCl223), the proportion of CD8^+^ T-cells in the infected lung significantly decreased after week 1 of infection by approximately 20% but then remained unchanged over the course of 100 days [9]. Depletion of CD8^+^ T-cells during this latent *C. neoformans* (UgCl223) infection did not affect lung CFUs, compared to isotype-treated mice, suggesting that CD8^+^ T-cells are not essential for controlling pulmonary fungal burden during latent infection [9]. In contrast, CD8^+^ T-cell depletion during the latent infection increased the amount of brain dissemination, suggesting these cells could be involved in the prevention of dissemination during latent infection [9].

### 2.2. Murine Cryptococcal Meningitis

CNS infiltrating CD8^+^ T-cells are present in the brain parenchyma and leptomeninges of *C. deneoformans* (NIH 52D) infected mice by day 8 post-infection and are maintained to at least day 15 [52]. When mice are depleted of CD8^+^ T-cells and then intravenously infected with *C. deneoformans* (145), survival is significantly worse compared to isotype control-treated mice, with a mean survival of 20.3 days compared to 26.4 days, respectively [53]. Despite the striking impact on mortality, CFUs from the lungs, spleen, kidney, liver, and brain were comparable between the CD8^+^ T-cell-depleted and isotype-control-treated mice [53]. These data imply that an immunological mechanism mediated by CD8^+^ T-cells influences disease outcomes during murine *C. deneoformans* (145) CM [53]. The activating receptor(s) and fungal ligand(s) involved in CD8^+^ T-cell sensing are currently unknown (Figure 1A). Additionally, the process by with CD8^+^ T-cells facilitate their anti-cryptococcal effects, presumably through cytotoxicity signaling pathways, remains to be explored (Figure 1A).

CD8^+^ T-cells have also been studied during CM in the context of vaccination. Immunization with cell culture filtrate followed by CD8^+^ T-cell depletion and challenge with *C. deneoformans* (184) resulted in comparable brain CFUs to infected control mice [34]. In another study investigating the impact of an immunizing *C. neoformans* (*sgl1*) mutant strain on survival from CM, Normile et. al. showed that depletion of both CD8^+^ T-cells and CD4^+^ T-cells prior to subsequent *C. neoformans* (H99) challenge led to extremely poor survival with 100% mortality from CM by ~ day 10 [54]. In comparison, CD8^+^ T-cell-depleted mice succumbed around 30 days post-infection [54]. Lastly, chitin deacetylase (Cda) -1 and -2 glucan-particle (GP) vaccination studies have shown that vaccinated CD8^+^ T-cell deficient β-2-microglubin^−/−^ knockout mice upon *C. neoformans* KN99 challenge have similar survival to wildtype vaccinated mice [55]. Similar results on CD8^+^ T-cells were seen in *cda∆1∆2∆3* vaccination studies as well [56]. Additionally, pulmonary CD8^+^ T-cells levels of GP-Cda1/Cda2 vaccinated wildtype mice peak by day 10 and upon ex vivo stimulation express IFN-γ, but not IL-17 and TNF-α [55]. Collectively, these data indicate that in the context of vaccination with Cda antigens, CD8^+^ T-cells are not important in long-term survival.

In contrast to localized pulmonary infection in mice, little is known regarding the phenotype, such as activation or residency status, and cytokine producing ability of CNS infiltrating CD8^+^ T-cells during murine CM.

### 2.3. Summary of Murine Studies

Murine studies demonstrate that CD8^+^ T-cells play a role in decreasing mortality from CM, likely through a combination of curtailing fungal dissemination from the lung to the CNS and immunomodulation. Additionally, during latent disease, CD8^+^ T-cells are involved in preventing dissemination to the brain. CD8^+^ T-cells are also a crucial source of IFN-γ during localized pulmonary and systemic disease and likely have cross talk with CD4^+^ T-cells, specifically T-helper (Th)-1 and Th17 cells, to influence IFN-γ production. The mechanism by which murine CD8^+^ T-cells sense *C. neoformans* and signal to perform cytotoxicity has not been explored, as demonstrated in Figure 1A. However, examination of human CD8^+^ T-cells has provided insight into the pathway involved in CD8^+^ T-cell cytotoxicity and cellular phenotype during antifungal and antiretroviral treatment.

### 2.4. Human PBMCs in Culture

Early studies on CD8^+^ T-cells isolated from healthy donors (HIV-negative) demonstrated that CD8^+^ T-cells can recognize acapsular *C. deneoformans Cap67* mutant in vitro and impair fungal growth [25,57,58]. IL-15 stimulation of CD8^+^ T-cells isolated from healthy donors also results in the impaired growth of *C. deneoformans* (*Cap67*) when compared to fungal cells alone or those cocultured with unstimulated CD8^+^ T-cells [58]. However, the activating receptor(s) and cryptococcal ligand(s) triggering CD8^+^ T-cell anti-cryptococcal effects are unknown (Figure 1B). In addition to impaired growth of fungal cells, CD8^+^ T-cells also significantly increase expression of the cytolytic protein granulysin [58]. Upon depletion of granulysin containing granules or gene interference, there is a reduction in the anti-cryptococcal effect of IL-15 stimulated CD8^+^ T-cells [58]. Collectively, these data propose that granulysin signaling is the likely pathway by which CD8^+^ T-cells impair fungal growth [58] (Figure 1B).

Other studies showed CD8^+^ T-cells can become activated by *C. deneoformans* (184A) cell culture concentrate, which is an aggregate of shedded capsule and cell wall components GXM, GalXM, and mannoprotein [25]. *C. deneoformans* (184A) cell culture concentrate is sufficient to activate CD8^+^ T-cells to induce shedding of endothelial cell adhesion molecule L-selectin, which is involved in cell extravasation [25]. It remains to be elucidated if the loss of L-selectin is involved in CD8^+^ T-cells becoming resident in *C. deneoformans* (184A) infected tissues.

Similar to murine CD8^+^ T-cells, human CD8^+^ T-cells have also been examined in the context of vaccination. To investigate the vaccine potential of human CD8^+^ T-cells, PBMCs were treated with a *C. deneoformans* (*Cap67*) cell wall prep and subsequently CD8^+^ T-cells were isolated and cultured with *C. deneoformans* (*Cap67*) to determine their anti-cryptococcal effects [58]. Pre-treated CD8^+^ T-cells retained their ability to impair the growth of *C. deneoformans* (*Cap67*), but their effect was reduced when CD4^+^ T-cells were depleted from the PBMC sample [58]. These results suggest that co-stimulation with CD4^+^ T-cells is a vital process in educating CD8^+^ T-cells to remember past pathogen exposures [58].

### 2.5. Human Cryptococcal Meningitis

At CM diagnosis, CD8^+^ T-cells predominate in the cerebrospinal fluid (CSF) over CD4^+^ T-cells and are present in the brain and leptomeninges at autopsy [52,59]. Over 75% of CNS infiltrating CD8^+^ T-cells are activated, as per co-expression of HLA-DR, and remain active over the course of 14 days of antifungal treatment [59]. Studies showed that CD8^+^ T-cells in the CSF express the homing receptors CXCR3 and CCR5, which are important for recruiting effector cells to inflamed tissues, and to recognize the ligands IP-10 and MIP-1α, respectively, among many others [60]. The proportion of CXCR3^+^ CCR5^+^ CD8^+^ T-cells increase significantly from baseline over the course of 14 days of antifungal treatment (~43% vs. 75%), implying active recruitment of CD8^+^ T-cells to the CNS [60].

In addition to antifungal treatment, patients with HIV and CM are also started on ART four to six weeks post-CM diagnosis to halt the replication of HIV and the destruction of CD4^+^ T-cells [61,62]. While ART improves CD4^+^ T-cell quantity and quality, ART also impacts CD8^+^ T-cells and there are several exceptional reviews discussing the topic [63,64]. While the proportion of peripheral activated HLA-DR^+^ CD38^+^ CD8^+^ T-cells and effector memory CD45RO^+^ CD8^+^ T-cells are maintained over the course of 12 weeks of ART initiation, the proportion of peripheral CXCR3^+^ CCR5^+^ CD8^+^ T-cells significantly decrease [60,65]. These data suggest that active effector CD8^+^ T-cells are being maintained in the periphery and fewer cells are being recruited to inflamed tissues during the early stages of ART treatment.

Delaying ART also reduces the risk of developing an over-exaggerated inflammatory response from rapid reconstitution of Th1 CD4^+^ T-cells, a condition known as immune reconstitution inflammatory syndrome (IRIS) [61,66,67]. In patients who develop IRIS, total CD8^+^ T-cells do not appear to increase when compared to CM diagnosis [59]. Several peripheral CD8^+^ T-cell subsets, such as antigen naïve CD45RO^−^ CD27^+^, central memory CD45RO^+^ CD27^+^, cytotoxic effector memory CD45RO^+^ CD27^−^, and terminally differentiated effector CD45RO^−^ CD27^−^ CD8^+^ T-cells are present at IRIS diagnosis and are comparable to levels in patients with CM alone [68]. However, upon ex vivo stimulation with GXM, central memory CD45RO^+^ CD27^+^ and cytotoxic effector memory CD45RO^+^ CD27^−^ peripheral CD8^+^ T-cell subsets from subjects with CM-IRIS have impaired production of IFN-γ, IL-2 and IL-17 compared to subjects with CM [68]. Together these findings indicate that, in the context of IRIS, cytokine production is negatively impacted even though CD8^+^ T-cells subset proportions do not change.

### 2.6. Summary of Human Studies

Though the mechanism of cytotoxicity has not been explored to date in murine studies, in vitro studies with CD8^+^ T-cells from healthy human donors has shown that CD8^+^ T-cells have an anti-cryptococcal effect. Similarly, in vitro studies have allowed for the discovery of granulysin as the cytolytic protein of interest utilized by human CD8^+^ T-cells at the microbial-immunological synapse. However, the activating receptor(s) on CD8^+^ T-cells and fungal ligand(s) which facilitate sensing as well as the intracellular signaling that triggers the release of granulysin are unknown.

Unlike in mouse models where CSF samples are difficult to obtain, the phenotype of human CD8^+^ T-cells in the CSF, and their relationship to cell subsets and chemokine receptor expression, have been extensively studied. Yet, despite our knowledge of human CD8^+^ T-cell phenotypes in the context of CM, ART usage, and IRIS, cytokine production has not been studied and therefore it is unclear how the CD8^+^ T-cells in the periphery and CNS are participating in shaping the overall immune environment during *C. neoformans* infection (Figure 1B). In contrast to murine and human CD8^+^ T-cells, the sensing, signaling, and cellular responses of NK cells to *C. neoformans* infection are better understood.

## 3. NK Cells in the Innate Immune Response

### 3.1. Murine Localized Pulmonary Infection

NK cell levels during murine localized pulmonary infection peak early in the disease course around three days post *C. neoformans* (clinical isolate YC-13) infection at 10% of total lung lymphocytes and appear to be partially dependent on monocyte chemoattractant protein (MCP)-1 to promote trafficking to the lungs [69]. Surprisingly, in the latent *C. neoformans* (clinical isolate UgCl223) infection model, NK cell proportions and cell counts do not change significantly over the course of 100 days; proportions remain similar to uninfected control mice [9].

Activating and inhibitory receptors on the surface of NK cells which facilitate or impair cytotoxicity, respectively, utilize intracellular adaptor signaling molecules, such as DAP12 among others. Considering their crucial role in intracellular signal transduction, studying DAP12 in knockout models has provided information on NK cell cytotoxic capacity. DAP12^−/−^ mice infected with *C. neoformans* (H99) have improved survival and reduced lung fungal burden compared to wildtype infected mice, in part due to a significant increase in NK cells in the lung [70]. Anti-cryptococcal activity of NK cells improves in the absence of DAP12 signaling adaptor, implying that DAP12 may be associated with inhibitory receptors that restrict cytotoxicity [70].

**Figure 2:**
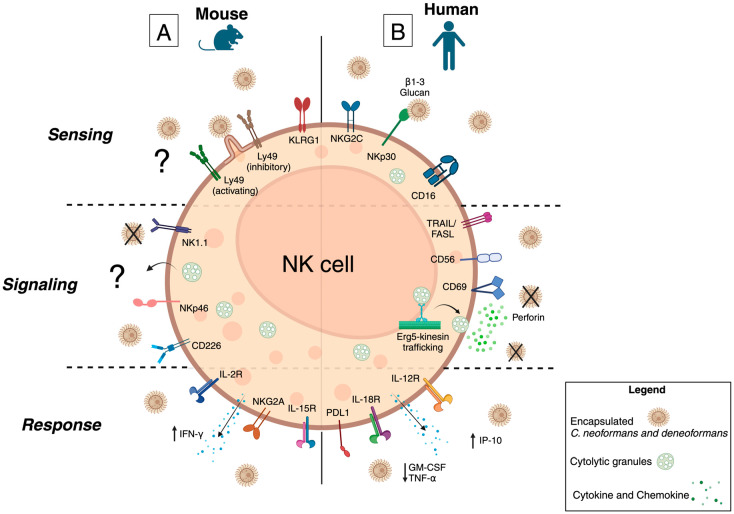
Murine and Human NK cell Sensing, Signaling, and Response to *C. neoformans* and *C. deneoformans.* Summary of the current body of literature regarding the mechanism of (**A**) murine and (**B**) human NK cell targeting, degranulation, and cytokine secretion after exposure to *C*. *neoformans* or *deneoformans*. (**A**) Murine NK cells interact with encapsulated *Cryptococcus* cells via “microvilli” or cellular protrusions, though the receptor or integrin interacting with the fungal cell capsule remains unknown. Though anti-cryptococcal activity of murine NK cells has been documented, the mechanism is undetermined but likely involves intracellular signaling pathways which are not mediated by DAP12 motifs. Murine NK cells are robust producers of the inflammatory cytokine IFN-γ during infection; the production of other inflammatory cytokines is unknown. (**B**) Human NK cell activating receptor NKp30 recognizes cryptococcal antigen β-1,3-glucan on the fungal cell wall which leads to the phosphorylation of intracellular tyrosine motifs by Src family kinases Fyn and Lyn and increase perforin transcription. Stimulation with IL-12 enhances NKp30 expression on NK cells from donors with HIV. The downstream signaling cascade remains to be elucidated. Granules containing granulysin and perforin are transported to the NK cell membrane via Erg5-kinesin. Perforin is released at the immunological synapse facilitating NK cell anti-cryptococcal effects and reducing fungal growth. However, it is unclear how perforin penetrates the *Cryptococcus* fungal cell wall. Upon exposure to Cryptococcus cells, NK cells increase secretion of chemoattractant IP-10 and reduce secretion of the inflammatory cytokine TNF-α and activating cytokine GM-CSF.

Through a combination of IL-2, IL-12, IL-15, and IL-18 cytokine stimulation, NK cells become active and can produce inflammatory cytokines, notably IFN-γ and TNF-α, which regulate downstream polarization of several cell types, including CD4^+^ T-cells and inflammatory macrophages [71]. NK cells in the lungs are one of the primary producers of IFN-γ during early *C. deneoformans* (B3501) infection, secondary only to T-cells [72] (Figure 2A). Depletion of NK cells led to a significant reduction in IFN-γ levels in the bronchiolar lavage fluid (BALF) of mice with *C. neoformans* (clinical isolate YC-11) infection compared to infected control mice [73].

Together these data suggest that NK cells play a role in IFN-γ secretion in the infected lung environment and that receptors utilizing DAP12 signaling adaptors are likely involved in recognition of *C. neoformans* cells, but the exact receptor(s) on murine NK cells have not been identified.

### 3.2. Murine Cryptococcal Meningitis

Though it has been established that murine NK cells are a vital source of inflammatory cytokine IFN-γ during cryptococcosis, there are conflicting data on the direct anti-cryptococcal effects of murine NK cells. Early transmission electron microscopy studies demonstrated NK cell “microvilli” interacting with the fungal cell capsule. However, the exact NK cell receptor(s) triggering this protrusion and the fungal capsule or cell wall ligand inducing it are unknown [74] (Figure 2A). When cultured with splenic NK cells, the growth of *C. deneoformans* (184A) was significantly reduced when compared to fungal cells alone [74].

Conversely, another group determined that NK cells, isolated from the spleen of SCID mice, did not destroy fungal cells—the CFUs were comparable to *C. neoformans* (clinical isolate YC-13) alone in culture [75]. The addition of stimulating cytokines IL-12 and IL-18 did not improve NK cell anti-cryptococcal ability. Instead, the proteins secreted by NK cells cultured with *C. neoformans* (clinical isolate YC-13) were able to activate peritoneal macrophages to significantly prevent fungal growth [75]. These data suggest that the killing activity of NK cells may be indirect through activation of other cell types.

### 3.3. Summary of Murine Studies

Murine studies demonstrate that NK cells are a vital source of IFN-γ which may impact downstream polarization of phagocytic cell populations. Though conflicting data exist on the ability of murine NK cells to sense *Cryptococcus*, many studies suggest that NK cells possess anti-cryptococcal abilities. However, the exact mechanism needs to be examined further. While intracellular signaling through DAP12 likely impairs NK cell cytotoxicity, the receptor(s) on NK cells and the fungal ligand(s) are unknown. In comparison to murine studies, studies on human NK cells have provided substantial evidence on cellular function during *C. neoformans* infection.

### 3.4. Human PBMCs in Culture

Initial studies using NK cells from healthy donors and NK cell cancer cell line YT established that NK cells can inhibit the growth of *C. deneoformans* (184A, 145, B3501, and *Cap67*), at various effector to target ratios [76,77,78,79,80]. The receptor ligand pair that allow NK cells to sense fungal cells were identified as activating receptor NKp30 and *C. neoformans* cell wall component β-1,3-glucan [81,82] (Figure 2B). When NKp30 on healthy donor NK cells engages with β-1,3-glucan, perforin is polarized to the site [81,82]. Likely due to the immunological dysfunction caused by HIV, NK cells from donors with HIV have decrease expression of NKp30 on the cell surface and impaired ability to inhibit the growth of *C. deneoformans* (B3501 and *Cap67)* when compared to NK cells from healthy donors [81,82,83]. Stimulation with activating cytokine IL-12 and then subsequent exposure to *C. deneoformans* (B3501) significantly improves NKp30 expression, anti-cryptococcal effects, and perforin release by NK cells from donors with HIV [81,82,83].

The mechanism by which NK cells impair the growth of *Cryptococcus* relies upon the cytolytic protein perforin [79,80,84,85] (Figure 2B). Upon exposure to *C. deneoformans* (B3501 and *Cap67*), NK cells significantly increase transcription of perforin, when compared to NK cells alone or to IL-2 and IL-12 stimulated cells [84]. Perforin polarization to the microbial-immunological synapse involves the phosphorylation of Src family kinases Fyn and Lyn, upstream of PI3K signaling pathway [80,82,85]. Inhibition of perforin release pathways or through gene silencing leads to the abolishment of the anti-cryptococcal activity of NK cells from healthy donors and YT cell line [79,80,84,86].

Trafficking of cytolytic granules, containing perforin or granulysin, within NK cells is facilitated by Erg5-kinesin; however, only perforin containing granules are released as determined by a significant decrease in intracellular concentrations of perforin compared to granulysin upon exposure to *C. deneoformans* (B3501) [87]. Lastly, perforin degranulation appears to be affected by environmental pH, as more acidic pHs enhance the ability of YT cells to impair the growth of *C. deneoformans* (B3501) and *C. neoformans* (H99) compared to physiological pH [86].

Exposure to *C. deneoformans* (B3501) reduces the ability of NK cells to secrete the inflammatory cytokines granulocyte-macrophage colony stimulating factor (GM-CSF) and TNF-α when compared to NK cells alone [78] (Figure 2B). Unfortunately, IFN-γ secretion was not investigated in human NK cells. Collectively, these data indicate that NK cells from healthy donors can prevent the growth of *C. deneoformans* through the release of perforin at the microbial-immunological synapse via the activating receptor NKp30 sensing cryptococcal ligand β-1,3-glucan. Chronic HIV reduces the expression of NKp30 on the cell surface and thus decreases anti-cryptococcal effects.

### 3.5. Human Cryptococcal Meningitis

Among NK cells, there are two primary cell subsets based on differentiation and cellular function: mature CD56^dim^ CD16^+^ cells primarily perform cytotoxicity, while immature CD56^dim^ CD16^−^ cells produce inflammatory cytokines such as TNF-α and IFN-γ, and the representation of each subset can influence the innate immune response during infection [71]. Over the course of 14 days of antifungal therapy, studies show significantly more mature CD56^dim^ NK cells in the blood and immature CD56^bright^ NK cells in the CSF of patients with CM [88]. CD69^+^ CD56^dim^ and CD69^+^ CD56^bright^ NK cell subsets were greater in the CSF compared to blood at hospitalization and Day 14 of antifungal treatment, implying that NK cell subsets in the CSF are active and remain active in the early days of antifungal treatment [88]. However, it is unclear if said activation is due to fungal antigens, the CSF cytokine environment, or a combination of both. When compared to peripheral CD56^bright^ NK cells, production of the chemokine IP-10 was greater while cytokine TNF-α was reduced in CSF infiltrating CD56^bright^ NK cells [88]. Again, IFN-γ expression was not measured.

During IRIS, it was reported that the percentage of NK cells in the CSF significantly decreases when compared to CM diagnosis, from 6.6% to 0.3%, respectively, though the proportion of CD56^bright^ and CD56^dim^ cells remained similar [59]. While the expression of the exhaustion marker PDL1 on CD56^bright^ NK cells did not change when patients developed IRIS, PDL1 expression on CD56^dim^ NK cells significantly increased indicating that CD56^dim^ NK cells in the CNS are potentially exhausted [59]. In studies of the bulk PBMC transcriptome of patients with IRIS, ingenuity pathway analysis of up- and down-regulated genes determined that genes involved in NK cells signaling were enriched in patients who develop IRIS 12-24 weeks after initiation of ART; only secondary to genes involved in Th1 and Th2 signaling [89]. Collectively these data imply that NK cells play an important role in the immune response long term during IRIS, but the functional ability of NK cells in the CNS remains to be examined.

### 3.6. Summary of Human Studies

Human NK cells utilize NKp30 activating receptor to sense fungal ligand β-1,3-glucan to engage in cytotoxicity through the release of perforin at the immunological synapse. NKp30 expression on healthy donors is higher when compared to NK cells from donors with HIV, yet this deficiency can be overcome with cytokine IL-12 stimulation. While studies on human NK cells show evidence of anti-cryptococcal effects, findings from murine studies are not as conclusive. This is potentially related to the receptor repertoire and functionality differences between murine and human NK cells [71]. Whereas intracellular signaling through DAP12 impairs murine NK cell function, it is unknown whether a similar mechanism occurs in human NK cells. Besides NKp30, additional studies need to be performed to determine if there are other activating receptors which sense *C. neoformans* or if inhibitory receptors play a role in impairing cytotoxicity.

Among NK cell subsets in the CSF, immature CD56^bright^ cells are greater in percentage compared to mature CD56^dim^ cells, which implies that secreting inflammatory cytokines may be an important role for CNS infiltrating NK cells. Similar to murine CD8+ T-cells in the CSF, the functional role of murine NK cells in the CSF and the subset breakdown is unclear. Lastly, the utility of alternative cytotoxicity pathways in fungal clearance, specifically ADCC and damage response signaling through TRAIL/FasL, have not been explored. While CD8^+^ T-cells and NK cells are the cytotoxic cell populations which have garnered up much of the attention in the field of *C. neoformans,* other cytotoxic cell populations such as γδT-cells, NK T-cells, and cytotoxic CD4+ T-cells have also been studied.

## 4. Lesser Studied Cytotoxic Cell Populations in Cryptococcosis

### 4.1. γδ T-Cells in Murine Pulmonary Infection

Though γδT-cells make up a small percentage of total leukocytes, γδT-cells are present during localized murine pulmonary *C. neoformans* (clinical isolate YC-13) infection and the percentage of cells peak approximately 6 days post-infection accounting for roughly 3% of total lymphocytes in the lung [69]. γδT-cells potentially may be detrimental in controlling fungal growth in the lung as in γTCR^−/−^ mice, or with depletion of γδT-cells during pulmonary *C. neoformans* (clinical isolate YC-13) infection, the lack of these cells leads to a reduction in pulmonary CFUs by day 14 of infection [69]. Additionally, depletion of γδT-cells leads to a significant increase in pulmonary and serum IFN-γ levels and no change in anti-inflammatory Th2 cytokines IL-4 and IL-10 when compared to isotype-control-treated mice [69]. However, others have noted conflicting findings related to the ability of γδT-cells to produce IFN-γ in the context of cryptococcosis. Pulmonary IFN-γ concentration were reduced in the BALF during γδT-cell depletion, in the context of IL-12 and IL-18 cytokine treatment of *C. neoformans* (clinical isolate YC-11) infected mice [73]. More studies are needed to clarify the relationship between γδT-cells and IFN-γ during murine pulmonary cryptococcosis.

γδT-cells have also been studied in IL-17^−/−^ mice vaccination experiments. γδT-cells are one of the main producers of IL-17A in the lung during *C. deneoformans* (B3501) pulmonary infection [90]. In IL-17RA^−/−^ mice deficient in neutrophils with pulmonary *C. neoformans* (H99γ) infection, studies show that γδT-cells were the main producer of IL-17A in the lung and was further confirmed ex vivo [91]. However, γδT-cell depletion did not impact pulmonary fungal burden, implying that γδT-cells may only be involved in polarization of T cells [91]. IL-17A is produced by γδT-cell during *C. neoformans* (H99) challenge after mutant *C. neoformans* (*Δsgl1*) vaccination and removal of γδT-cells abrogates protection from disseminated *C. neoformans* (H99) post vaccination [92].

To date, the cryptococcal ligand recognized by the γδTCR-cell receptor or other activating receptors is unknown. It is unclear if γδT-cells have direct anti-cryptococcal effects via cytotoxicity and, if so, which cytolytic proteins are involved in the process. Lastly, murine pulmonary studies have not been translated to humans and no formal studies have been published on γδT-cells in humans with CM.

### 4.2. NK T-Cells in Murine Pulmonary Infection

Vα14^+^ NK T-cells (NK T-cells) levels in the murine lung during localized pulmonary *C. neoformans* (clinical isolate YC-13) infection peaks at 7-days post-infection at a proportion of 3.5% of the total pulmonary lymphocytes and partially relies on the MCP-1 chemoattractant signal for localization [69,93]. These NK T-cells are a crucial source of IFN-γ, as *C. neoformans* (clinical isolate YC-13) infected NK T-cell deficient mice have significantly less pulmonary IFN-γ compared to infected control mice [93]. NK T-cell deficient mice have higher pulmonary CFUs 7-, 14-, and 21-days post-infection compared to infected control mice [93]. Interestingly, NK T-cells in the murine lung are not strong producers of IL-17A or IFN-γ during *C. deneoformans* (B3501) infection, suggesting there may be strain or species differences that influence NK T-cell function [90].

In terms of cytotoxic ability, in vitro and ex vivo assays of murine or human NK T-cells have not been performed; therefore, the contribution of NK T-cells to fungal clearance is unknown. Similar to γδT-cells, little is known about the relevance of these cells in humans and no studies have been published on NK T-cells in human CM.

### 4.3. Cytotoxic CD4^+^ T-Cells in Human C. neoformans Infection

Studies show that bulk human CD4^+^ T-cells can prevent the growth of acapsular *C. deneoformans* (*Cap67*) in vitro in an IL-2 dependent manner [94]. The cytolytic protein involved in this process is possibly granulysin, as mRNA levels of granulysin increased upon culture with acapsular *C. deneoformans* (*Cap67*) [94]. It should be noted, however, that the development of and phenotype of cytotoxic CD4^+^ T-cells have been robustly characterized in the decades since these initial studies were performed in *C. deneoformans* [95]. Therefore, it is difficult to evaluate the previous study using bulk CD4^+^ T-cells. With the ability now to fluorescently sort cytotoxic CD4^+^ T-cells based on extracellular receptor repertoire, revisiting past experiments could provide information on the role of human cytotoxic CD4^+^ T-cells during cryptococcosis. Studies on murine cytotoxic CD4^+^ T-cells during pulmonary and disseminated cryptococcal disease have not been performed.

## 5. Future Directions

As discussed, there are a considerable number of questions to be investigated regarding cytotoxic cell populations during murine and human *C. neoformans* infection. To assist with addressing these research questions, innovative cellular technologies are being introduced to the Cryptococcus research community including T-cell receptor engineering, *C. neoformans*-specific tetramers, single-cell and bulk-RNA sequencing of host immune and fungal cells, and murine inducible knockout systems, to name a few.

One such tool is the *C. neoformans* latent infection model with a clinical isolate. We can now study immune cell function in an immunocompetent host in a clinically relevant manner that is similar to human latent infection [9]. Characterizing the phenotype and function of cytotoxic cell populations in a latent infection model can provide information on cellular responses and potential targets for immunomodulation. Similarly, investigations into engineering CD8^+^ T-cells with enhanced ability to target *C. neoformans,* through the addition of GXM-specific chimeric antigen receptor, are underway [96,97,98,99]. This *C. neoformans*-specific CD8^+^ T-cell adjuvant host directed therapy could be delivered alongside antifungals and antiretrovirals to improve the host immune response. Other fields have used similar combinations of therapeutic approaches for treatment of autoimmune and oncological diseases [100,101].

## 6. Conclusions

Evidence accumulated over several decades has demonstrated that cytotoxic cell populations play an important role in fungal clearance during localized pulmonary infection and systemic disease. Murine and human studies on CD8^+^ T-cells have shown that CD8^+^ T-cells utilize a granulysin secreting cytotoxicity pathway at the microbial-immunological synapse and that CD8^+^ T-cells are a vital source of IFN-γ in infected tissues. On the contrary, NK cells utilize activating receptor NKp30 to sense β-1,3-glucan fungal ligand that elicits cytotoxicity and degranulation of perforin containing granules. Considering the breath of diversity among activating and inhibitory receptor genes, there remains much to be studied regarding sensing of fungal cells and receptor signaling in CD8^+^ T-cells and NK cells [40,102]. Investigations of lesser-known cytotoxic populations, such as γδT-cells, NK T-cells, and cytotoxic CD4^+^ T-cells are in their infancy and research into the clinical relevance and usefulness of these cell populations during infection are warranted. With improvements in flow cytometry and mass cytometry technologies, the potential exists to clearly identify rare cell populations, such as these lesser-known cytotoxic cells, even with a limited clinical sample.

In conclusion, research into cytotoxic cell populations during *C. neoformans* infection is a niche ripe for exploration, and as our knowledge on cellular function improves, there is long-term potential to improve patient morbidity and mortality.

## Data Availability

Not applicable.

## References

[1] Pirofski L.A., Casadevall A. (2020). The state of latency in microbial pathogenesis. J. Clin. Investig..

[2] Alanio A. (2020). Dormancy in Cryptococcus neoformans: 60 years of accumulating evidence. J. Clin. Investig..

[3] Goldman D.L., Khine H., Abadi J., Lindenberg D.J., Pirofski L.-A., Niang R., Casadevall A. (2001). Serologic Evidence forCryptococcus neoformansInfection in Early Childhood. Pediatrics.

[4] Garcia-Hermoso D., Janbon G., Dromer F. (1999). Epidemiological evidence for dormant Cryptococcus neoformans infection. J. Clin. Microbiol..

[5] Saha D.C., Goldman D.L., Shao X., Casadevall A., Husain S., Limaye A.P., Lyon M., Somani J., Pursell K., Pruett T.L. (2007). Serologic evidence for reactivation of cryptococcosis in solid-organ transplant recipients. Clin. Vaccine Immunol..

[6] Singh N., Dromer F., Perfect J.R., Lortholary O. (2008). Immunocompromised Hosts: Cryptococcosis in Solid Organ Transplant Recipients: Current State of the Science. Clin. Infect. Dis..

[7] Domaica C.I., Fuertes M.B., Uriarte I., Girart M.V., Sardanons J., Comas D.I., Di Giovanni D., Gaillard M.I., Bezrodnik L., Zwirner N.W. (2012). Human natural killer cell maturation defect supports in vivo CD56(bright) to CD56(dim) lineage development. PLoS ONE.

[8] Brunet K., Alanio A., Lortholary O., Rammaert B. (2018). Reactivation of dormant/latent fungal infection. J. Infect..

[9] Ding M., Smith K.D., Wiesner D.L., Nielsen J.N., Jackson K.M., Nielsen K. (2021). Use of Clinical Isolates to Establish Criteria for a Mouse Model of Latent Cryptococcus neoformans Infection. Front. Cell Infect. Microbiol..

[10] Ellis J., Bangdiwala A.S., Cresswell F.V., Rhein J., Nuwagira E., Ssebambulidde K., Tugume L., Rajasingham R., Bridge S.C., Muzoora C. (2019). The Changing Epidemiology of HIV-Associated Adult Meningitis, Uganda 2015–2017. Open Forum Infect. Dis..

[11] Hakim J.G., Gangaidzo I.T., Heyderman R.S., Mielke J., Mushangi E., Taziwa A., Robertson V.J., Musvaire P., Mason P.R. (2000). Impact of HIV infection on meningitis in Harare, Zimbabwe: A prospective study of 406 predominantly adult patients. AIDS.

[12] Jarvis J.N., Meintjes G., Williams A., Brown Y., Crede T., Harrison T.S. (2010). Adult meningitis in a setting of high HIV and TB prevalence: Findings from 4961 suspected cases. BMC Infect. Dis..

[13] Rajasingham R., Govender N.P., Jordan A., Loyse A., Shroufi A., Denning D.W., Meya D.B., Chiller T.M., Boulware D.R. (2022). The global burden of HIV-associated cryptococcal infection in adults in 2020: A modelling analysis. Lancet Infect. Dis..

[14] Rajasingham R., Smith R.M., Park B.J., Jarvis J.N., Govender N.P., Chiller T.M., Denning D.W., Loyse A., Boulware D.R. (2017). Global burden of disease of HIV-associated cryptococcal meningitis: An updated analysis. Lancet Infect. Dis..

[15] Farrar J., Rajasingham R., Rolfes M.A., Birkenkamp K.E., Meya D.B., Boulware D.R. (2012). Cryptococcal Meningitis Treatment Strategies in Resource-Limited Settings: A Cost-Effectiveness Analysis. PLoS Med..

[16] May R.C., Stone N.R.H., Wiesner D.L., Bicanic T., Nielsen K. (2015). Cryptococcus: From environmental saprophyte to global pathogen. Nat. Rev. Microbiol..

[17] Litvintseva A.P., Carbone I., Rossouw J., Thakur R., Govender N.P., Mitchell T.G. (2011). Evidence that the Human Pathogenic Fungus Cryptococcus neoformans var. grubii May Have Evolved in Africa. PLoS ONE.

[18] Mukaremera L., Lee K.K., Wagener J., Wiesner D.L., Gow N.A.R., Nielsen K. (2018). Titan cell production in cryptococcus neofromans reshapes the cell wall and capsule composition during infection. Cell Surf..

[19] Oscar Zaragoza M.L.R., De Jesus M., Frases S., Dadachova E., Casadevall A. (2009). Chapter 4 The Capsule of the Fungal Pathogen Cryptococcus neoformans. Adv. Appl. Microbiol..

[20] Baker L.G., Specht C.A., Lodge J.K. (2011). Cell Wall Chitosan Is Necessary for Virulence in the Opportunistic Pathogen Cryptococcus neoformans. Eukaryot. Cell.

[21] Reese A.J., Yoneda A., Breger J.A., Beauvais A., Liu H., Griffith C.L., Bose I., Kim M.J., Skau C., Yang S. (2006). Loss of cell wall alpha(1–3) glucan affects Cryptococcus neoformans from ultrastructure to virulence. Mol. Microbiol..

[22] Decote-Ricardo D., LaRocque-de-Freitas I.F., Rocha J.D.B., Nascimento D.O., Nunes M.P., Morrot A., Freire-de-Lima L., Previato J.O., Mendonça-Previato L., Freire-de-Lima C.G. (2019). Immunomodulatory Role of Capsular Polysaccharides Constituents of Cryptococcus neoformans. Front. Med..

[23] Monari C., Paganelli F., Bistoni F., Kozel T.R., Vecchiarelli A. (2008). Capsular polysaccharide induction of apoptosis by intrinsic and extrinsic mechanisms. Cell. Microbiol..

[24] LaRocque-de-Freitas I.F., Rocha J.D.B., Nunes M.P., Oliveira P.A.V., Nascimento D.d.O., Freire-de-Lima L., Takiya C.M., Morrot A., Decote-Ricardo D., Previato J.O. (2018). Involvement of the capsular GalXM-induced IL-17 cytokine in the control of Cryptococcus neoformans infection. Sci. Rep..

[25] Dong Z.M., Jackson L., Murphy J.W. (1999). Mechanisms for induction of L-selectin loss from T lymphocytes by a cryptococcal polysaccharide, glucuronoxylomannan. Infect. Immun..

[26] Doitsh G., Greene W.C. (2016). Dissecting How CD4 T Cells Are Lost During HIV Infection. Cell Host Microbe.

[27] Chen B. (2019). Molecular Mechanism of HIV-1 Entry. Trends Microbiol..

[28] Schaefer M.R., Wonderlich E.R., Roeth J.F., Leonard J.A., Collins K.L. (2008). HIV-1 Nef targets MHC-I and CD4 for degradation via a final common beta-COP-dependent pathway in T cells. PLoS Pathog..

[29] Jarvis J.N., Casazza J.P., Stone H.H., Meintjes G., Lawn S.D., Levitz S.M., Harrison T.S., Koup R.A. (2013). The Phenotype of the *Cryptococcus*-Specific CD4+ Memory T-Cell Response Is Associated With Disease Severity and Outcome in HIV-Associated Cryptococcal Meningitis. J. Infect. Dis..

[30] Mukaremera L., Nielsen K. (2017). Adaptive Immunity to *Cryptococcus neoformans* Infections. J. Fungi.

[31] Lindell D.M., Moore T.A., McDonald R.A., Toews G.B., Huffnagle G.B. (2006). Distinct Compartmentalization of CD4+ T-Cell Effector Function Versus Proliferative Capacity during Pulmonary Cryptococcosis. Am. J. Pathol..

[32] Huffnagle G.B., Yates J.L., Lipscomb M.F. (1991). Immunity to a pulmonary Cryptococcus neoformans infection requires both CD4+ and CD8+ T cells. J. Exp. Med..

[33] Hill J.O. (1992). CD4+ T cells cause multinucleated giant cells to form around Cryptococcus neoformans and confine the yeast within the primary site of infection in the respiratory tract. J. Exp. Med..

[34] Buchanan K.L., Kozel T.R., Doyle H.A. (2000). Requirement for CD4+T Lymphocytes in Host Resistance againstCryptococcus neoformansin the Central Nervous System of Immunized Mice. Infect. Immun..

[35] Lim T.S., Murphy J.W. (1980). Transfer of immunity to cryptococcosis by T-enriched splenic lymphocytes from Cryptococcus neoformans-sensitized mice. Infect. Immun..

[36] Elsegeiny W., Marr K.A., Williamson P.R. (2018). Immunology of Cryptococcal Infections: Developing a Rational Approach to Patient Therapy. Front. Immunol..

[37] Onyishi C.U., May R.C. (2022). Human immune polymorphisms associated with the risk of cryptococcal disease. Immunology.

[38] Mohamed S.H., Nyazika T.K., Ssebambulidde K., Lionakis M.S., Meya D.B., Drummond R.A. (2022). Fungal CNS Infections in Africa: The Neuroimmunology of Cryptococcal Meningitis. Front. Immunol..

[39] Meya D.B., Williamson P.R. (2024). Cryptococcal Disease in Diverse Hosts. N. Engl. J. Med..

[40] Pende D., Falco M., Vitale M., Cantoni C., Vitale C., Munari E., Bertaina A., Moretta F., Del Zotto G., Pietra G. (2019). Killer Ig-Like Receptors (KIRs): Their Role in NK Cell Modulation and Developments Leading to Their Clinical Exploitation. Front. Immunol..

[41] Barrow A.D., Martin C.J., Colonna M. (2019). The Natural Cytotoxicity Receptors in Health and Disease. Front. Immunol..

[42] Zhang N., Bevan M.J. (2011). CD8+ T Cells: Foot Soldiers of the Immune System. Immunity.

[43] Shiromizu C.M., Jancic C.C. (2018). γδ T Lymphocytes: An Effector Cell in Autoimmunity and Infection. Front. Immunol..

[44] Cenerenti M., Saillard M., Romero P., Jandus C. (2022). The Era of Cytotoxic CD4 T Cells. Front. Immunol..

[45] Ochoa M.C., Minute L., Rodriguez I., Garasa S., Perez-Ruiz E., Inoges S., Melero I., Berraondo P. (2017). Antibody-dependent cell cytotoxicity: Immunotherapy strategies enhancing effector NK cells. Immunol. Cell Biol..

[46] Cassioli C., Baldari C.T. (2022). The Expanding Arsenal of Cytotoxic T Cells. Front. Immunol..

[47] Prager I., Watzl C. (2019). Mechanisms of natural killer cell-mediated cellular cytotoxicity. J. Leukoc. Biol..

[48] Lindell D.M., Moore T.A., McDonald R.A., Toews G.B., Huffnagle G.B. (2005). Generation of antifungal effector CD8+ T cells in the absence of CD4+ T cells during Cryptococcus neoformans infection. J. Immunol..

[49] Lindell D.M., Ballinger M.N., McDonald R.A., Toews G.B., Huffnagle G.B. (2006). Diversity of the T-Cell Response to PulmonaryCryptococcus neoformansInfection. Infect. Immun..

[50] Murdock B.J., Huffnagle G.B., Olszewski M.A., Osterholzer J.J. (2014). Interleukin-17A enhances host defense against cryptococcal lung infection through effects mediated by leukocyte recruitment, activation, and gamma interferon production. Infect. Immun..

[51] Neal L.M., Qiu Y., Chung J., Xing E., Cho W., Malachowski A.N., Sandy-Sloat A.R., Osterholzer J.J., Maillard I., Olszewski M.A. (2017). T Cell–Restricted Notch Signaling Contributes to Pulmonary Th1 and Th2 Immunity during Cryptococcus neoformans Infection. J. Immunol..

[52] Chrétien F., Lortholary O., Kansau I., Neuville S., Gray F., Dromer F. (2002). Pathogenesis of Cerebral Cryptococcus neofromans Infection after Fungemia. J. Infect. Dis..

[53] Mody C.H., Chen G.-H., Jackson C., Curtis J.L., Toews G.B. (1994). Un vivo depletion of murine CD8 positive T cells impairs survival during infection with a highly virulent strain ofCryptococcus neoformans. Mycopathologia.

[54] Normile T.G., Rella A., Del Poeta M. (2021). Cryptococcus neoformans Δsgl1 Vaccination Requires Either CD4+ or CD8+ T Cells for Complete Host Protection. Front. Cell. Infect. Microbiol..

[55] Wang R., Oliveira L.V.N., Lourenco D., Gomez C.L., Lee C.K., Hester M.M., Mou Z., Ostroff G.R., Specht C.A., Levitz S.M. (2023). Immunological correlates of protection following vaccination with glucan particles containing Cryptococcus neoformans chitin deacetylases. Npj Vaccines.

[56] Specht C.A., Wang R., Oliveira L.V.N., Hester M.M., Gomez C., Mou Z., Carlson D., Lee C.K., Hole C.R., Lam W.C. (2024). Immunological correlates of protection mediated by a whole organism, Cryptococcus neoformans, vaccine deficient in chitosan. mBio.

[57] Syme R.M., Wood C.J., Wong H., Mody C.H. (1997). Both CD4+ and CD8+ human lymphocytes are activated and proliferate in response to Cryptococcus neoformans. Immunology.

[58] Ma L.L., Spurrell J.C., Wang J.F., Neely G.G., Epelman S., Krensky A.M., Mody C.H. (2002). CD8 T cell-mediated killing of Cryptococcus neoformans requires granulysin and is dependent on CD4 T cells and IL-15. J. Immunol..

[59] Meya D.B., Okurut S., Zziwa G., Rolfes M.A., Kelsey M., Cose S., Joloba M., Naluyima P., Palmer B.E., Kambugu A. (2015). Cellular immune activation in cerebrospinal fluid from ugandans with cryptococcal meningitis and immune reconstitution inflammatory syndrome. J. Infect. Dis..

[60] Chang C.C., Omarjee S., Lim A., Spelman T., Gosnell B.I., Carr W.H., Elliott J.H., Moosa M.-Y.S., Ndung’u T., French M.A. (2013). Chemokine Levels and Chemokine Receptor Expression in the Blood and the Cerebrospinal Fluid of HIV-Infected Patients With Cryptococcal Meningitis and Cryptococcosis-Associated Immune Reconstitution Inflammatory Syndrome. J. Infect. Dis..

[61] Boulware D.R., Meya D.B., Muzoora C., Rolfes M.A., Huppler Hullsiek K., Musubire A., Taseera K., Nabeta H.W., Schutz C., Williams D.A. (2014). Timing of antiretroviral therapy after diagnosis of cryptococcal meningitis. N. Engl. J. Med..

[62] World Health Organization (2022). Guidelines for Diagnosing, Preventing and Managing Cryptococcal Disease among Adults, Adolescents and Children Living with HIV.

[63] Warren J.A., Clutton G., Goonetilleke N. (2019). Harnessing CD8+ T Cells Under HIV Antiretroviral Therapy. Front. Immunol..

[64] Perdomo-Celis F., Taborda N.A., Rugeles M.T. (2019). CD8+ T-Cell Response to HIV Infection in the Era of Antiretroviral Therapy. Front. Immunol..

[65] Bayiyana A., Okurut S., Nabatanzi R., Zziwa G., Boulware D.R., Lutwama F., Meya D. (2019). Longitudinal Changes in Cd4(+), Cd8(+) T Cell Phenotype and Activation Marker Expression Following Antiretroviral Therapy Initiation among Patients with Cryptococcal Meningitis. J. Fungi.

[66] Neal L.M., Xing E., Xu J., Kolbe J.L., Osterholzer J.J., Segal B.M., Williamson P.R., Olszewski M.A. (2017). CD4(+) T Cells Orchestrate Lethal Immune Pathology despite Fungal Clearance during Cryptococcus neoformans Meningoencephalitis. mBio.

[67] Delliere S., Guery R., Candon S., Rammaert B., Aguilar C., Lanternier F., Chatenoud L., Lortholary O. (2018). Understanding Pathogenesis and Care Challenges of Immune Reconstitution Inflammatory Syndrome in Fungal Infections. J. Fungi.

[68] Meya D.B., Okurut S., Zziwa G., Cose S., Boulware D.R., Janoff E.N. (2019). HIV-Associated Cryptococcal Immune Reconstitution Inflammatory Syndrome Is Associated with Aberrant T Cell Function and Increased Cytokine Responses. J. Fungi.

[69] Uezu K., Kawakami K., Miyagi K., Kinjo Y., Kinjo T., Ishikawa H., Saito A. (2004). Accumulation of γδ T Cells in the Lungs and Their Regulatory Roles in Th1 Response and Host Defense against Pulmonary Infection with Cryptococcus neoformans. J. Immunol..

[70] Heung L.J., Hohl T.M. (2016). DAP12 Inhibits Pulmonary Immune Responses to Cryptococcus neoformans. Infect. Immun..

[71] Abel A.M., Yang C., Thakar M.S., Malarkannan S. (2018). Natural Killer Cells: Development, Maturation, and Clinical Utilization. Front. Immunol..

[72] Yamamoto H., Nakamura Y., Sato K., Takahashi Y., Nomura T., Miyasaka T., Ishii K., Hara H., Yamamoto N., Kanno E. (2014). Defect of CARD9 leads to impaired accumulation of gamma interferon-producing memory phenotype T cells in lungs and increased susceptibility to pulmonary infection with Cryptococcus neoformans. Infect. Immun..

[73] Qureshi M.H., Zhang T., Koguchi Y., Nakashima K., Okamura H., Kurimoto M., Kawakami K. (1999). Combined effects of IL-12 and IL-18 on the clinical course and local cytokine production in murine pulmonary infection with Cryptococcus neoformans. Eur. J. Immunol..

[74] Murphy J.W., Hidore M.R., Nabavi N. (1991). Binding interactions of murine natural killer cells with the fungal target Cryptococcus neoformans. Infect. Immun..

[75] Kawakami K., Koguchi Y., Qureshi M.H., Yara S., Kinjo Y., Uezu K., Saito A. (2013). NK Cells Eliminate Cryptococcus neoformans by Potentiating the Fungicidal Activity of Macrophages Rather than by Directly Killing Them upon Stimulation with IL-12 and IL-18. Microbiol. Immunol..

[76] Murphy J.W., Hidore M.R., Wong S.C. (1993). Direct interactions of human lymphocytes with the yeast-like organism, Cryptococcus neoformans. J. Clin. Investig..

[77] LEVITZ S.M., P.DUPONT M., SMAIL E.H. (1994). Direct Activity of Human T Lymphocytes and Natural Killer Cells against *Cryptococcus neoforman*. Infect. Immun..

[78] Murphy J.W., Zhou A., Wong S.C. (1997). Direct Interaction of Human Natural Killer Cells with *Cryptococcus neofromans* inhibits Granulocytes-macrophage colony-stimulating Factor and Tumor Necrosis Factor Alpha Production. Infect. Immun..

[79] Ma L.L., Wang C.L.C., Neely G.G., Epelman S., Krensky A.M., Mody C.H. (2004). NK Cells Use Perforin Rather than Granulysin for Anticryptococcal Activity. J. Immunol..

[80] Wiseman J.C.D., Ma L.L., Marr K.J., Jones G.J., Mody C.H. (2007). Perforin-Dependent Cryptococcal Microbicidal Activity in NK Cells Requires PI3K-Dependent ERK1/2 Signaling. J. Immunol..

[81] Li S.S., Kyei S.K., Timm-McCann M., Ogbomo H., Jones G.J., Shi M., Xiang R.F., Oykhman P., Huston S.M., Islam A. (2013). The NK receptor NKp30 mediates direct fungal recognition and killing and is diminished in NK cells from HIV-infected patients. Cell Host Microbe.

[82] Li S.S., Ogbomo H., Mansour M.K., Xiang R.F., Szabo L., Munro F., Mukherjee P., Mariuzza R.A., Amrein M., Vyas J.M. (2018). Identification of the fungal ligand triggering cytotoxic PRR-mediated NK cell killing of *Cryptococcus* and *Candida*. Nat. Commun..

[83] Kyei S.K., Ogbomo H., Li S., Timm-McCann M., Xiang R.F., Huston S.M., Ganguly A., Colarusso P., Gill M.J., Mody C.H. (2016). Mechanisms by Which Interleukin-12 Corrects Defective NK Cell Anticryptococcal Activity in HIV-Infected Patients. mBio.

[84] Marr K.J., Jones G.J., Zheng C., Huston S.M., Timm-McCann M., Islam A., Berenger B.M., Ma L.L., Wiseman J.C., Mody C.H. (2009). Cryptococcus neoformans directly stimulates perforin production and rearms NK cells for enhanced anticryptococcal microbicidal activity. Infect. Immun..

[85] Oykhman P., Timm-McCann M., Xiang R.F., Islam A., Li S.S., Stack D., Huston S.M., Ma L.L., Mody C.H. (2013). Requirement and redundancy of the Src family kinases Fyn and Lyn in perforin-dependent killing of Cryptococcus neoformans by NK cells. Infect. Immun..

[86] Islam A., Li S.S., Oykhman P., Timm-McCann M., Huston S.M., Stack D., Xiang R.F., Kelly M.M., Mody C.H. (2013). An acidic microenvironment increases NK cell killing of Cryptococcus neoformans and Cryptococcus gattii by enhancing perforin degranulation. PLoS Pathog..

[87] Ogbomo H., Timm-McCann M., Barnes T., Xiang R.F., Jamil K., Ganguly A., Stack D., Huston S.M., Li S.S., Colarusso P. (2018). Granule-Dependent NK Cell Killing of Cryptococcus Requires Kinesin to Reposition the Cytolytic Machinery for Directed Cytotoxicity. Cell Rep..

[88] Naranbhai V., Chang C.C., Durgiah R., Omarjee S., Lim A., Moosa M.-Y.S., Elliot J.H., Ndung’u T., Lewin S.R., French M.A. (2014). Compartmentalization of innate immune responses in the central nervous system during cryptococcal meningitis/HIV coinfection. Aids.

[89] Louis I.V.-S., Chang C.C., Shahid S., French M.A., Bohjanen P.R. (2018). Transcriptomic Predictors of Paradoxical Cryptococcosis-Associated Immune Reconstitution Inflammatory Syndrome. Open Forum Infect. Dis..

[90] Sato K., Yamamoto H., Nomura T., Kasamatsu J., Miyasaka T., Tanno D., Matsumoto I., Kagesawa T., Miyahara A., Zong T. (2020). Production of IL-17A at Innate Immune Phase Leads to Decreased Th1 Immune Response and Attenuated Host Defense against Infection with Cryptococcus deneoformans. J. Immunol..

[91] Wozniak K.L., Kolls J.K., Wormley F.L. (2012). Depletion of neutrophils in a protective model of pulmonary cryptococcosis results in increased IL-17A production by gamma/delta T cells. BMC Immunol..

[92] Normile T.G., Chu T.H., Sheridan B.S., Del Poeta M. (2022). Vaccine protection by Cryptococcus neoformans Deltasgl1 is mediated by gammadelta T cells via TLR2 signaling. Mucosal Immunol..

[93] Kawakami K., Kinjo Y., Uezu K., Yara S., Miyagi K., Koguchi Y., Nakayama T., Taniguchi M., Saito A. (2001). Monocyte Chemoattractant Protein-1-Dependent Increase of Vα14 NKT Cells in Lungs and Their Roles in Th1 Response and Host Defense in Cryptococcal Infection. J. Immunol..

[94] Zheng C.F., Ma L.L., Jones G.J., Gill M.J., Krensky A.M., Kubes P., Mody C.H. (2006). Cytotoxic CD4+ T cells use granulysin to kill Cryptococcus neoformans, and activation of this pathway is defective in HIV patients. Blood.

[95] Hoeks C., Duran G., Hellings N., Broux B. (2022). When Helpers Go Above and Beyond: Development and Characterization of Cytotoxic CD4+ T Cells. Front. Immunol..

[96] da Silva T.A., Hauser P.J., Bandey I., Laskowski T., Wang Q., Najjar A.M., Kumaresan P.R. (2021). Glucuronoxylomannan in the Cryptococcus species capsule as a target for Chimeric Antigen Receptor T-cell therapy. Cytotherapy.

[97] Dos Santos M.H., Machado M.P., Kumaresan P.R., da Silva T.A. (2021). Titan Cells and Yeast Forms of Cryptococcus neoformans and Cryptococcus gattii Are Recognized by GXMR-CAR. Microorganisms.

[98] Dos Santos M.H., Machado M.P., Kumaresan P.R., da Silva T.A. (2022). Modification of Hinge/Transmembrane and Signal Transduction Domains Improves the Expression and Signaling Threshold of GXMR-CAR Specific to *Cryptococcus* spp.. Cells.

[99] Machado M.P., Dos Santos M.H., Guimaraes J.G., de Campos G.Y., Oliveira Brito P.K.M., Ferreira C.M.G., Rezende C.P., Frota N.F., Soares S.G., Kumaresan P.R. (2023). GXMR-CAR containing distinct GXM-specific single-chain variable fragment (scFv) mediated the cell activation against Cryptococcus spp. And had difference in the strength of tonic signaling. Bioengineered.

[100] Schett G., Mackensen A., Mougiakakos D. (2023). CAR T-cell therapy in autoimmune diseases. Lancet.

[101] Jogalekar M.P., Rajendran R.L., Khan F., Dmello C., Gangadaran P., Ahn B.C. (2022). CAR T-Cell-Based gene therapy for cancers: New perspectives, challenges, and clinical developments. Front. Immunol..

[102] Pazina T., Shemesh A., Brusilovsky M., Porgador A., Campbell K.S. (2017). Regulation of the Functions of Natural Cytotoxicity Receptors by Interactions with Diverse Ligands and Alterations in Splice Variant Expression. Front. Immunol..

